# A scoping review examining the integration of exercise services in clinical oncology settings

**DOI:** 10.1186/s12913-022-07598-y

**Published:** 2022-02-21

**Authors:** Elochukwu F. Ezenwankwo, Daniel A. Nnate, Godspower D. Usoro, Chimdimma P. Onyeso, Ijeoma B. Anieto, Sam C. Ibeneme, Yumna Albertus, Victoria E Lambert, Antoninus O. Ezeukwu, Ukachukwu O. Abaraogu, Delva Shamley

**Affiliations:** 1 Centre for Health Through Physical Activity, Lifestyle and Sports (HPALS), Department of Human Biology, Faculty of Health Sciences, University of Cape Town, Cape Town, South Africa; 2 Cancer Research Initiative, Faculty of Health Sciences, University of Cape Town, Anzio Road, ObservatoryCape Town, 7925 South Africa; 3 Department of Nursing and Community Health, School of Health and Life Sciences, Glasgow Caledonian University, Glasgow, UK; 4 Countess of Chester Hospital NHS Foundation Trust, Health Park, Liverpool Road, Chester, CH2 1UL UK; 5 Department of Medical Rehabilitation, Faculty of Health Sciences and Technology, College of Medicine, University of Nigeria, Enugu Campus, Enugu, Nigeria; 6 Department of Physiotherapy, Faculty of Health Sciences, School of Therapeuitc Studies, University of the Witwatersrand, 7 York Road, Parktown, Johannesburg, 2193 South Africa; 7 Department of Physiotherapy, Faculty of Health Sciences and Technology, King David University of Medical Sciences, Ebonyi State, Uburu, Nigeria; 8 Department of Physiotherapy and Paramedicine, School of Health and Life Sciences, Glasgow Caledonian University, Glasgow, UK; 9 Clinical Research Centre, Faculty of Health Sciences, University of Cape Town, Cape Town, South Africa

**Keywords:** Exercise-based rehabilitation, Service integration, Reach, Adoption, Cost, Utilization, Cancer care

## Abstract

**Background:**

Addressing questions surrounding the feasibility of embedding exercise service units in clinical oncology settings is imperative for developing a sustainable exercise-oncology clinical pathway. We examined available literature and offered practical recommendations to support evidence-based practice, policymaking, and further investigations.

**Methods:**

Four thousand eight hundred sixty-three unique records identified in Embase, CINAHL, MEDLINE, Web of Science Core Collection, and ProQuest (Health and Medicine) were screened for studies that recruited cancer patients, assessed the co-location of exercise service and cancer treatment units, and reported findings on service implementation. Evidence from six studies providing data from over 30 programs was integrated using narrative synthesis.

**Results:**

Service implementation was relatively modest across the included studies. Exercise services were delivered by physiotherapists, exercise physiologists, and kinesiologists and funded mainly through grants and private donations, with staff salaries accruing as the major expense. Service penetration, adoption, and acceptability were generally low. However, studies recorded high clinician/patient satisfaction. Major barriers to service integration were limited funding, lack of detailed implementation plan, and low organizational buy-in. Common reasons for non-utilization, missed sessions, and dropouts were lack of interest, unwellness, hospital readmission, disease progression, and adverse skeletal events.

**Conclusion:**

Implementing exercise services in clinical oncology settings seems an effective approach for increasing access to exercise-based rehabilitation for individuals on cancer treatment. While this model appears feasible for patients/clinicians, efforts are required to optimize service integration both in the short and long term. Key priorities include seeking [local] actions to address issues relating to funding and organizational buy-in. Important considerations may include developing an implementation plan to guide the implementation process, expanding the patient core management team to include staff from the exercise rehabilitation unit, and exploring the role of patient feedback in increasing clinician participation (e.g., treating oncologists and nurses) in the referral process. Future research should consider effective strategies to promote patients’ sense of self-efficacy and behavioral control and, further, the place of audit and feedback in improving exercise service delivery and overall service implementation.

**Supplementary Information:**

The online version contains supplementary material available at 10.1186/s12913-022-07598-y.

## Introduction

From in vitro models, early preclinical studies, and large population-based observational studies to high-quality clinical exercise efficacy trials and behavior change studies involving ‘real world’ scenarios, the cancer exercise literature abounds with clear and profound evidence of the mitigating effects and health benefits of exercise in the trajectory of cancer care [1–7]. Engaging in regular exercise program not only is safe and feasible for cancer patients but can also improve treatment tolerance [8–10], facilitate early recovery [8–10], and reduce the length of hospital stay [11, 12]. There is also evidence that exercise can slow cancer progression [13], lower the risk of recurrence, readmission, and mortality [13, 14]. An improved exercise lifestyle has also shown great promise for a better quality of life [10, 15], particularly early return to work and other day-to-day activities [10]. Recent reports suggest that only about 30% to 47% of cancer patients are meeting current global exercise recommendations [16, 17]. While many factors preclude cancer patients from engaging in regular exercise, lack of access to exercise-based rehabilitation as part of routine care in treatment settings has remained a major barrier [10, 18].

Calls to make exercise-based rehabilitation an integral component of routine oncology care are gaining more traction globally as the World Health Organization moves to increase *global access to high-quality rehabilitation as an essential healthcare service* for individuals with chronic disease [10]. Many stakeholders are increasingly acknowledging a foremost implication of this ‘call to action’ to include embedding exercise services in cancer treatment settings [14]. Oncology care models that foster integrative exercise-cancer care units may provide a more pragmatic approach for delivering access to timely, flexible, and high-quality exercise-based rehabilitation to cancer patients. When patients are offered early access to individualized and supervised exercise programs, they are well-positioned to develop the physical, mental and psychosocial capacities to confront the challenges associated with cancer treatment even before they set in. Providing access to exercise-based rehabilitation within a cancer care setting is likely to encourage integrated and multidisciplinary oversight, creating opportunities for routine joint patient evaluation, shared decision making, and triage. A key benefit of this approach is that oncology clinicians, including doctors, nurses, and exercise specialists, can recognize any potential risks/threats and intervene more holistically and timeously. This approach is likely to increase patients’ confidence and satisfaction in their care. As exercise adoption and maintenance are particularly challenging in posttreatment populations, an integrated exercise-oncology care model may be the greatest leverage available to healthcare providers to intervene most critically within the window of time when patients are more amenable to behavior change [19].

Embedding an exercise service unit in a typical cancer treatment setting may present some challenges to patients, clinicians, and the health service system. First, the actual process of installing an exercise unit within existing treatment settings may require slight to huge (infra)structural (re)adjustments. The likely potential for disruption in workflow could come at a risk to patients as they may be unable to access routine care more efficiently during such time. Second, many health systems are currently grappling with underfunding globally. Hence even where integrative exercise-oncology models are less resource-intensive, health services may find it challenging to hire exercise specialists with the right credentials and experience for handling the peculiar exercise needs and challenges of cancer patients. Another critical factor is the capacity for patient screening, triage, and referral. As this is a relatively new frontier, the present clinical oncology workforce may lack the clarity, culture, and the will to assess, advise, and rightly refer patients for exercise medicine [14]. Many facilities lack robust guideline-concordant care with well-defined and streamlined patient screening/evaluation algorithms and referral pathways [8]. Together, these concerns raise a question about the feasibility, including the cost implications and sustainability of implementing an exercise service unit in a standard oncology clinical setting.

## Methods

### Research objective

We aim to provide a comprehensive summary of peer-reviewed literature on the feasibility of implementing an exercise service unit within a cancer treatment setting. To achieve this, we performed a scoping review of the literature using the modified framework of Levac and colleagues [20]. The current review does not warrant consent to participate or institutional ethics approval as only publicly available peer-reviewed literature was utilized, with no primary data collection [20]. However, we reported our findings using the Preferred Reporting Items for Systematic reviews and Meta-Analyses extension for Scoping Reviews (PRISMA-ScR) guidelines [21].

### Study eligibility

We included studies that evaluated the implementation of exercise service units within cancer care settings in this review. To be eligible, service units were to have a well-defined structure and be located in a clinical setting (e.g., inpatient or outpatient services, public and private practice). Essentially, studies must report data for one of the following implementation outcomes: acceptability, adoption, appropriateness, practicality (including cost), feasibility, fidelity, penetration, sustainability, and quality assessment. As such, studies including trials evaluating exercise benefits in cancer populations were excluded. We also included studies providing stakeholder perspectives on the co-location of exercise service and cancer care units. No restrictions were placed on study design or publication date. Non-primary research, including reviews, commentaries, and viewpoint articles, including non-English studies were further excluded.

### Information sources and search

EE and DN conducted a comprehensive literature search on Embase via Ovid, CINAHL via EBSCOhost, MEDLINE via Ovid, Web of Science Core Collection via Clarivate Analytics, and ProQuest (Health and Medicine) independently. In developing the search strategy, relevant search terms and medical subject headings (MeSH) were identified by exploring the National Library of Medicine Database [22] and, further, by reviewing a recent review of exercise interventions for cancer survivors [23]. Specific keywords and MeSH terms applied in the search include (but are not limited to) cancer, neoplasm, exercise, feasibility, etc., and implementation outcomes such as acceptability, adoption, appropriateness, practicality, etc (See Additional File 1). Additionally, recent systematic and meta-analytic reviews of cancer exercise literature were scanned for relevant citations.

### Article screening and selection

Identified records were exported to RefWorks software for de-duplication and then Microsoft Excel Spreadsheet for screening. EE and DN independently screened the titles and abstracts of all retrieved citations and, further, the full texts of the remaining articles using the review’s eligibility criteria. Differences in opinions during the screening process were resolved by discussion in consultation with GU.

### Data extraction and analysis

A data extraction form was developed and tested to guide data extraction. First, we reviewed varieties of constructs as considered in the Implementation Outcome Framework of Proctor and Colleagues [24], Bowen’s framework [25], and the Reach, Effectiveness, Adoption, Implementation, and Maintenance (RE-AIM) framework of Glasgow and colleagues [26], Next, we adapted a list of priority outcomes drawing on recent evidence and our experience in implementation research (Table 1). Data on study characteristics, cancer care setting, nature/components of exercise services, and implementation outcomes were extracted. This review focused on the following key implementation outcomes: implementation, cost, reach/penetration, service uptake/adoption, acceptability, patient satisfaction, fidelity, and sustainability. Quantitative and qualitative results were extracted, analyzed, and integrated to produce the final synthesis.

**Table 1:** Operationalization of implementation outcomes

**Outcomes**	**Definition**	**Measurement metrics**
Reach/Penetration	The absolute representativeness of individuals, including healthcare providers and patients, and organizations who are willing to utilize exercise services integrated as part of cancer care	• Total number of referrals for exercise-based rehabilitation relative to the total eligible patient population
Service uptake/adoption	Service utilization by an organization as evidenced by reports on the total number of staff referring patients for exercise-based rehabilitation	• Number of patient referrers
Acceptability	The extent to which exercise services is deemed suitable, satisfactory, and attractive to the patients or the healthcare providers	• Number of accepted referrals • Service compliance (including attrition) • Adverse events
Patient satisfaction	The extent to which exercise services is deemed satisfactory by the patients	• Documented reports on patient satisfaction
Implementation	The extent to which exercise-based rehabilitation can be delivered to the intended population successfully	• Workforce • Equipment • Service promotion • Referral mechanism/pathway • Program structure • Session duration • Funding
Cost	The cost implications of service implementation	• Salaries • Purchase cost • Delivery cost
Fidelity	The degree of service providers’ compliance with existing pre-implementation plan and recommendation guidelines	• Documented efforts including strategies to ensure fidelity including consistency of service delivery
Sustainability	The extent to which exercise services becomes institutionalized as a standard in routine cancer care	• [infra]structural adjustments • Increased workforce • Increased funding

## Results

### Study description

**Fig. 1:**
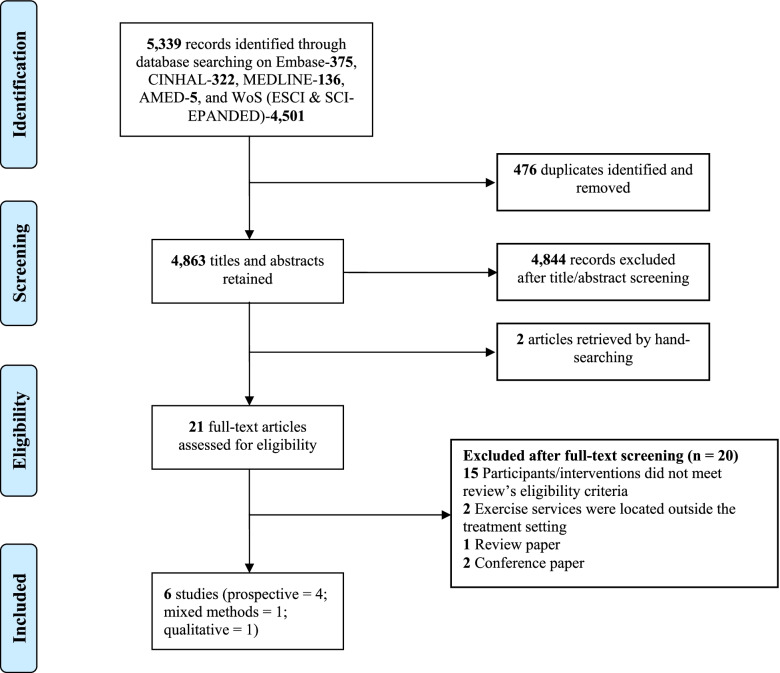
PRISMA flow diagram of the study selection procedure

Six studies providing data from over 30 exercise programs were included in this review [27–32]. Details of the screening and selection process are provided in Fig. 1. One of the studies was conducted in Canada [31] and the rest were carried out in Australia [27–30, 32]. Included studies were largely prospective, involving varying cancer types and patient demographics except for Dennett et al., [28]— a qualitative report on clinicians’ perspectives. Patients were generally above 50 years and on active treatment with either chemotherapy, radiation therapy, or immunotherapy. Of the oncology services, two were publicly funded [27, 31], one was privately funded [29, 30], and another involved both public and private hospitals/cancer centers [32]. Exercise services were individualized and group-based and largely featured a combination of aerobic and resistance exercise [27, 29–32]. More details on the included studies are provided in Table 2.

**Table 2:** Description of included studies

**Author** **Country**	**Study**	**Population**	**Healthcare setting**	**Exercise service**	**Service description**	**Implementation outcomes**
Dennett 2021 [27]; Dennett 2021 [28] Australia	**Design:** Prospective pre-post designQualitative exploration **Evaluation:** 6 months	Adult cancer survivors (n = 64) currently receiving or preparing for cancer treatment (curative or palliative) admitted as an inpatient or outpatient Age: 63 ± 11yrs Gender: Male: n = 41; 56%; Female: n = 32, 44%	Cancer unit — inpatient oncology ward + outpatient day oncology center offering chemotherapy — embedded in a publicly funded tertiary hospital	Exercise-based rehabilitation within a hospital-based cancer treatment center	Individually tailored, physiotherapist-led group-based circuit exercise class **Frequency**: 1-2x/wk **Duration:** 8 weeks	All
Kennedy 2020 [29]; Newton 2020 [30] Australia	**Design:** Retrospective evaluation **Evaluation:** 50 months	Individuals (n = 73) receiving radiation therapy and/or chemotherapy **Median age:** 58.5; IQR: 48-67) **Gender:** Female: n = 43, 67.2%; male: n = 21, 32.8% **Cancer type:** Breast: n = 39, 60.9%; Prostate: n = 13, 20.3%; Colorectal: n = 3, 4.7%; Lung: n = 3, 4.7%; Other: n = 6, 9.4% **Secondary cancer report:** n = 3, 4.8% **Treatment type:** Radiation: n = 55, 85.9%; Chemo: n = 4, 6.3%); Radio + chemo: n = 5, 7.8%	Private oncology care clinic (GenesisCare) providing primarily outpatient-based radiation therapy and medical oncology treatments	Exercise service Clinic (Co-LEC) established in 2013 by researchers from Edit Cowan University, in partnership with GenesisCare	Patient tailored (progressive)/group-based resistance (2-3 sets; 6-12 reps) + aerobic exercise (20mins; 60%-80% estimated HRmax) delivered by an AEP **Frequency:** 60mins/session; 2-3x/wk **Duration:** Throughout treatment course (Average: 13wks)	All
Dalzell 2017 [31] Canada	**Design:** Prospective **Evaluation:** 60 months	234 new and follow-up cancer patients e.g., sample demographics for sample 2 months evaluation (multiple cancer types;) Mean Age: 52 ± 15.5yrs Female: 65% Patients on active treatment: 52% Patients with advanced disease or metastatic cancer: 35.5% Bone metastasis: 16% Bone metastasis: 16%	Integrated oncology and palliative care center within a publicly funded general hospital	Multimodal rehabilitation care model with hospital-based exercise oncology referral component (ActivOnco) embedded in a cancer center	Individualized plus group-based multicomponent exercise with patient education, exercise counseling, and self-management	All but sustainability and cost
Dennett 2017 [32] Australia	Design: Ex post facto design using mixed methods approachEvaluation: 2 wks	Patients with different cancer diagnoses, disease stages, and treatment status	Public and private hospitals/cancer centers across 6 states/territories	31 eligible programs identified from 56 public settings and 9 private settings	Individualized exercise program (Block = 14 programs; rolling = 17 programs) comprising mainly a combination of aerobic, resistance, and flexibility exercise 6-10 patients/session **Frequency/duration:** Outpatient: 2x/wk for 8wks; inpatient: 2x/day for the duration of inpatient stay (~ 2 wks)	All but sustainability and cost

Note: *AEP* Accredited exercise physiologist, *wk* week

### Summary of implementation

A summary of the implementation outcomes is provided in Table 3.

**Table 3:** Summary of implementation outcomes

	**Dennett 2021** [27]; **2021** [28]	**Kennedy 2020** [29]; **Newton 2020** [30]	**Dalzell 2017** [31]	**Dennett 2017** [32]
Implementation	**Workforce** **Employed clinical staff:** 1 Senior physiotherapist (20hr/wk) 1 Mid-level physiotherapist (19.5hr/wk) **Support staff:** 1 senior research physiotherapist **Service operation** **Resources:** physiotherapy gym with existing equipment **Average wait time to the first appointment:** 20 days (range 0–99) **Average time taken for first appointment:** 51 min (SD 7) **Service access:** 4d/wk (Mon-Thur) **Attendance option**: 1x or 2x/wk (Ihr/session) **Clinician to patient ration (per group class):** 1:4 **Service promotion:** Within and outside health facility** (e.g., **flyer, poster, newsletter) with the aid of the organization’s communications officer **Referral mechanism:** Direct verbal referral (i.e., in-person, telephone); use of centralized email address (i.e., by including patient name/contact details); self-referralClinicians were encouraged to have a brief conversation on exercise with patients prior to referrals. **Transition plan:** Patients were referred to community-based rehab, existing sub-acute multidisciplinary Cancer rehab, home-based rehab, and occupational therapy	**Workforce:** 4 AEPs including consultants (AEPs were separate to the patient core care team) **Service operation:** Independent of the cancer center: patient triage and integrated medical record were lacking **Resources:** Provided by ECU **Service access:** 3days/wk; 2hrs/session with lack of co-ordination between gym and treatment times **Service promotion:** Not reported **Referral mechanism**Pathway: direct verbal referrals from clinicians; self-referralReferrals were made only when oncologists remembered and had the time	**Workforce: **5 physiotherapists (I clinical director and 4 staff physiotherapists) + 3 kinesiologists with training and experience in oncology **Service operation:** Independent of the cancer center **Resources:** Provided by Hope and Care **Service promotion:** Presentations on the values of exercise interventions to various departments **Referral mechanism:** well-defined patient triage and referral pathwaysSources include oncologists, allied health workers, self-referral, other sources including wellness centers **Transition plan:** Home-based exercise program, wellness center	Workforce: Physiotherapy: 21/31 programs; Exercise Physiology: 20/31 programs **Service promotion:** Exercise fliers, letters to GPs, community awareness programs **Service structure:** outpatient programs: 2x/wk for 8 wks; inpatient programs: 2x/day for the duration of inpatient stay (approximately 2 weeks) Early morning sessions were less practical and received the lowest patient attendance Developing flexible and rolling program is critical to enhancing practicality **Referral mechanism** Patient feedback to their primary doctors was a key driver of more referrals from doctors **Transition plan:** Home-based exercise program, community groups
Cost	**Funding:** External service improvement grant **Cost to patient:** no cost **Health service** Staffing, e.g., payment of salaries: AUD $160,916 **Consumables:** Mobile phone costs (AUD $180; $30 per month)Printing of assessment forms and home exercise programs (5 pages per patient x 73 patients @ 0.66 c /page) (AUD $2) **Total Cost:** AUD $161,098 **Cost to health service per patient:** AUD $1,104	**Funding:** ECU research grant **Cost to patient:** no cost Operational cost was covered through a research grant	**Funding: P**rivate donations	**Funding sources:** public = 14; private = 17
Reach/Penetration	~10% of patients treated in the cancer center (i.e., 155 referrals including self-referrals)	12% (i.e., 237 out of 1963 patients that received cancer treatment over a 50-month period) Average annual reach = 10-14%	1635 patients over a 5-year evaluation period, with an average of 5.8 follow-up visits	31 eligible programs identified from 46 public hospitals/cancer centers and 39 private hospitals/centers across 6 out of 8 states/territories
Service uptake	46 staff made 148 referrals over the 6 months evaluation period: medical: n = 32, 22%; nurses: n = 53, 36%; allied health: n = 63, 43% **Facilitators of service utilization:** Service visibility, convenience, building rapport, accessibility, timing, and staff experience	Number of oncologists with at least 1 patient attending Co-LEC = 11/11 **Sources of referrals:** oncologists = 21%; nurses = 20%	Referrals were largely from oncologists (35%) and nurses (36%) (e.g., over a 2-month referral period)	Referral sources: oncologists (28/31 programs); allied health clinicians (21/31 programs) Poor knowledge among doctors on the role of exercise in cancer management was a major limiting factor
Acceptability	44% (52* out of eligible 119 patients) **Refused referrals**: n = 67, 43% [Reasons: not interested (n = 17), unsure (n = 16), unwell/treatment related (n = 3), work (n = 2), location/parking (n = 2), home-based exercise (21) other (n = 6)] **No. of refusals after 1st session:** n = 2 (reason: readmission = 1) **Compliance:** 38 patients elected for 2x/wk with 56% completing 7/16 sessions; 14 patients elected for 1x/wk with 40% completing 3/8 sessions **Missed sessions were due to:** Refusal (25%) Unwell due to treatment (23%) **Drop out:** n = 20; 38% (Reasons: COVID-19 restrictions; hospital readmission, disease progression)	27% (i.e., 64 out of 237 referrals over a 50 month) Common reason for non-service utilization was lack of awareness of its availability	71% compliance (over 3 years) in a sample of 41 patients with multiple myeloma (81% had bone lesion) on active treatment **Dropouts**: Increased with the incidence of skeletal-related events, including pathologic fracture, spinal cord compression, and radiation for stabilization of bone lesions	**Overall, annual enrolment per program:** 10-70 patients; 2000 survivors per year across Australia
Satisfaction	n = 57^#^, 100%) Access (timing, facility, location): n = 46, 81% Willingness to recommend others to participate during treatment: n = 57, 100% Feeling of improved overall health/wellbeing: n = 56, 98% **Sources of dissatisfaction** Difficulties with access: n = 6, 8% Difficulties were largely due to lack of parking space	Social value: n = 11 out of 61 patients Improved treatment experience: 12 out of 61 patients Positivity: 24/61 patients Staff experience/professionalism: 17/61 patients **Sources of dissatisfaction** Lack of coordination between treatment and gym times: 33/51 patients Parking issues: 5/51 patients Lack of transition plan at the end of the program: 4/51 patients	—	**Patient cantered:** programs addressed individual patient needs and goals Programs increased opportunities for social support Sources of dissatisfaction Program timing (attendance were lowest for early morning sessions) Parking issues Travel distances particularly for metropolitan centers
Fidelity	Exercise service was implemented by clinicians with 5.5 years oncology-specific experience and prior cancer-specific training in acute and community cancer settings. A steering committee comprising a consumer, clinical directors, physiotherapy manager and a community partner ensured service implementation Program staff and other hospital physiotherapists received three 1hr education sessions on cancer and rehabilitation Medical, nursing, and allied health staff received 3 presentations to provide updates throughout program implementation	Service implementation was spearheaded by 3 AEPs with experience in exercise oncology	Continuous staff mentoring and education	—
Sustainability	Philanthropic funds were sought to pay staff salaries to sustain the program beyond the pilot period	**Funding:** Direct clinical operational cost was covered by ECU and GenesisCare to support service continuation at the end of the feasibility phase **Structural adjustments (mainly due to inadequate funds):** Operational hours reduced to 2hrs/wk (1hr/2days/wk) Eligibility was rescinded for patients receiving chemotherapy alone Service duration was reduced to 3 months for all patients regardless of treatment duration **Challenges** Communication gap between ECU and GenesisCare Financial model was lacking— Co-LEC was not generating revenue	—	—

Note: *AEP* Accredited exercise physiologist, *ECU* Edith Cowan University, *Co-LEC* Co-located exercise clinic, *wk* week

### Service implementation

Exercise services were largely operated independently of the housing treatment settings, and program staff was generally not part of the patient core care team [27, 29, 31, 32]. Exercise programs were delivered by physiotherapists [27, 31, 32], exercise physiologists [29, 32], and kinesiologists [31] experienced in oncology settings. One study reported a clinician-to-patient ratio of 1:4 [27]. Access to exercise services varied across the included studies. In one study, exercise sessions were available every Monday to Thursday, and participants had access to a one-hour gym session once or twice a week [27]. In another study, participants had access to three exercise sessions per week, with each session lasting two hours [29]. In Dennett et al., [32] outpatients accessed programs twice a week for eight weeks while inpatients attended up to two sessions per day for the entire duration of their hospital stay. Early morning sessions and lack of coordination between treatment and gym times were reported as key barriers to program access [29, 32]. Structured patient referral mechanism was generally lacking except for one study that showed evidence of a well-designed patient triage and referral pathways [31]. Referrals were largely verbal, from the oncologist and other healthcare providers directly to the exercise programs [27, 29, 32]. Self-referrals were also reported in all the included studies [27, 29, 31, 32]. One study reported using an email system to create a central access point for the clinicians [27]. Exercise programs were promoted differently across the included studies. Strategies such as flyers [27, 32], posters [27], newsletters [27], letters to general practitioners [32], community awareness [32], and in-hospital presentations [31] were adopted to promote the programs within and outside the health facilities. At program completion, patients were largely recommended for home-based exercise programs [27, 31, 32], a hospital-based multidisciplinary rehabilitation program [27] or community-based rehabilitation programs [27, 31, 32].

### Cost

Exercise programs were delivered at no cost to the patients; however, operational costs were largely covered with public funds, including grants [27, 29] and private donations [31]. Staff salaries accrued a greater part of the operational cost [27, 29]. In one study, the per-patient cost to the health service within the evaluation period was AUD $1,104 [29].

### Service reach

Program reach as reported in two studies was 10% [27] and 12% [29], with Kennedy et al. [29] reporting an annual reach of 10% to 14% over a 50-month evaluation period. In one study,(31) 1635 patients were evaluated in 5 years with an average of 5.8 follow-up visits. Another study identified only 31 programs from 85 public and private hospitals/cancer centers in 6 out of 8 states/territories in Australia [32].

### Service uptake

Individual referral data were generally lacking. In one study, 46 staff made 148 referrals over a 6-month evaluation period [27]. In another study, all the oncologists (*n* = 11) consulting in the cancer center had at least one patient under their management attending exercise clinic within the 50-month evaluation period [29]. Referrals were largely from doctors, nurses, and allied health staff [27, 29, 31, 32]. Referrals from nurses were around 20% [29] and 36% [27, 31] of the total referrals. Referrals from doctors were generally poor —i.e., 21%–22% [27, 29] and 35% [31]. Factors that improved service uptake among clinicians were patient feedback, regular service promotion, enhanced visibility, convenience, building rapport among the clinicians (treating oncologists, nurses and exercise specialists), accessibility, good timing, and staff experience [27, 28, 32]. Poor knowledge among doctors on the role of exercise in cancer management was reported as a major barrier to service uptake [32].

### Acceptability

Two studies reported 27% [27] and 44% [29] acceptance rates. One study reported 71% compliance in a sample of 41 patients over three years [31]. In another study, 56% of the participants who elected for three weekly exercise sessions attended 7 out of 16 sessions [27]. In the same study, 40% of the participants electing for once per week exercise sessions attended only 3 out of 8 sessions [27]. A different study reported 10% to 70% annual enrolment per program (*n* = 31 programs), averaging 2000 cancer survivors per year across Australia [32]. Common reasons for non-utilization, missed sessions, and dropout were COVID-19 restrictions [27], hospital readmission [27], disease progression [27], lack of awareness of service availability [29], adverse skeletal events,[31] unwellness due to treatment [27], and patient refusal [27].

### Patient satisfaction

Patient satisfaction was high amid varying cancer types and patient demographics. In one study, 81% of the total responders (*n* = 46) were satisfied with the facility, location, and timing of the program, and all the responders (*n* = 57) reported their willingness to refer other patients to the program during treatment [27]. Key drivers of patient satisfaction were improved wellbeing and overall treatment experience [27, 29], staff experience and professionalism [29], social value [27, 29, 32], feeling of empowerment [29], and patient-centered service [32]. Wrong program timing [32], lack of coordination between gym and treatment times [29], parking issues [27, 29, 32], travel distance [32], and lack of transition plan [29] were frequently mentioned as major causes of dissatisfaction.

### Fidelity

None of the studies provided reports on service fidelity. However, to ensure a high standard of care, service implementation was largely led by clinicians with experience in oncology settings [27, 29, 31, 32]. In one study, this was further ensured by a steering committee comprising a patient, clinical directors, physiotherapy manager, and a community partner [27]. Other approaches maintained to ensure a high-quality service delivery include regular updates [27] and continuous staff mentoring and education [31].

### Sustainability

To sustain exercise services beyond the evaluation period, philanthropic funds were sought to pay staff salaries in one study [27]. In another study [29], program duration was reduced to three months besides partnering with the cancer care center to cover operational costs and scaling down the operational hours to two days per week (one hour per session). Eligibility was further rescinded for patients receiving chemotherapy alone in the same study [29]. Reported lack of a financial model and effective communication between partnering organizations were the major threats to the program's sustainability [29].

## Discussion

The impetus for the current review stems primarily from the growing need to close the research-practice gap that has long existed in the field of exercise oncology. Even with the overwhelming evidence on the feasibility, safety, and clinical benefits of exercise in cancer patients, exercise-based rehabilitation is still generally considered an adjunct instead of an integral component of care during treatment. The result of this evidence-practice gap is that most patients do not have access to exercise services while receiving cancer treatment, a period when the debilitating effects of cancer treatments are at their peak and can best be mitigated or ameliorated with exercise-based rehabilitation [8, 14, 33]. Despite a limited number of studies, implementing exercise services in [proximity to] a cancer unit appears to be an effective approach for increasing access to exercise-based rehabilitation for individuals on active treatment [27–32]. While this approach seems to be feasible for both the clinicians (the referring clinicians and those delivering the programs) and patients, the current evidence is not a confirmation of the overall feasibility of exercise service integration in oncology care settings. The lack of a clear implementation plan was evident across the included studies [27–32]. As critical to a successful service implementation as this may be, issues relating to funding and organizational buy-in hold even far greater implications for effective service integration and long-term sustainability.

Overall, service implementation was modest even though fidelity to any pre-implementation plan was not demonstrated. As a direct consequence of this downside, capacity for patient screening and risk stratification, effective patient triage, and structured referral mechanisms were generally lacking. Exercise services were largely operated independently of the clinical settings in which they were embedded, and staff leading these programs were also not part of patient core management team [29, 32]. This compromised the potential for shared decision-making in most programs and enabled communication gaps between the clinical staff and exercise service providers [29, 32]. Lack of an implementation plan was implicated in the poor coordination between exercise sessions and treatment time. In one study, patients reported that they could not attend exercise sessions because they constantly clashed with their treatment times [29]. As co-location does not automatically translate to successful service integration, a detailed implementation plan ensures that structures and strategies that reflect the changing dynamics of the clinical environment housing an exercise service unit are put in place to drive effective and sustainable integration.

Access to exercise programs was relatively feasible and similar across the included studies. Most programs were open to participants two to three times a week [27, 29, 31, 32]. In one study, for example, exercise services were available from Monday to Thursday during the six months evaluation period [27]. In another study, patients on admission had daily access to exercise programs throughout their inpatient stay [32]. Another consistent finding across the included studies was the simplified and convenient nature of the referral process [27, 29, 31, 32]. Although well-defined referral pathways were generally lacking, patient referrals were simple and convenient. Exercise referrals were mostly verbal, directly from the referring clinicians (the oncologists, nurses, and other allied health staff) to the exercise programs [27, 29, 32]. One study reported an additional use of a central access point (email referral) to facilitate patient referrals further [27]. Another major facilitator of referrals was patient feedback [27, 28, 32]. One study reported that doctors who received positive feedback directly from their patients were more inclined to refer more patients to the exercise program [28]. By encouraging patients to feedback to their clinicians on their thoughts, experiences, and concerns with the exercise program, exercise service providers can motivate doctors [and nurses] to engage more fully and proactively with the referral process. Barriers to patient referrals were recorded at both individual and health service levels. At the individual level, while most doctors were aware of exercise benefits, particularly during cancer treatment, many lacked the will to refer patients to exercise programs. In one study, doctors reported referring patients to the exercise program only when they remembered and had the time to do so [29]. At the health service level, low organizational buy-in, even with the reported evidence of adequate service promotion, was a major finding [27, 29, 31, 32].

Lack of organizational buy-in may be responsible for the overall low service penetration and utilization among the clinicians. For example, two studies reported overall service reach ranging between 10% and 12% [27, 29], with Kennedy et al. [29] reporting an annual reach of 10% to 14% over a 50-month evaluation period. One study identified only 31 exercise service programs in the whole of 6 out of 8 states/territories in Australia [32]. Successful integration of exercise services in routine oncology care demands a concerted effort to develop and identify the right implementation strategies to provoke a cultural shift in the host organization, which is critical for increasing organizational buy-in. One way to achieve this is by providing education to the healthcare providers working in oncology settings. Healthcare providers can only refer patients to exercise service programs if they know how, when, and where patients can be referred for such services [8]. To refer patients for exercise services, doctors and nurses, for example, should understand and appreciate the rehabilitation dimensions of their patient care and effectively and proactively screen patients for exercise interventions [8, 33]. As this is a relatively new frontier in cancer care, many healthcare providers in oncology settings may need to be trained on how to use exercise screening algorithms and referral guidelines to adopt these tools [8, 33]. Tools such as electronic medical records and integrated/central referral systems can improve service ease and efficiency, and ultimately utilization [8, 33].

Organizational buy-in can also be improved by enhancing the visibility of the service units. In one study, the referring clinicians were pleased with the value created by the frequent presence of physiotherapists in the cancer unit as they actively featured in ward rounds, offered clinical insights even during informal discussions, and took part in patient assessment and decision making [28]. Another strategy to increase service utilization is to increase staff confidence, particularly in the safety of the exercise programs [28]. This can be achieved by ensuring that the physiotherapists and other exercise specialists working in these settings have the right skills and training to match the peculiarities and dynamics of cancer care. Specifically, exercise prescriptions must be based on well-established international guidelines and recommendations while reflecting patients’ circumstances, needs, preferences, and values [10]. Good communication, knowledge sharing, patient responsiveness, and teamwork can foster strong relationships between staff leading exercise programs and oncology clinicians and ultimately enhance service utilization [28]. Regular service promotion within the clinical setting is another strategy to increase organizational buy-in. One study achieved this by providing regular updates and timely reminders through staff presentations, use of newsletters, and by introducing an alerting system in electronic medical records [27, 28, 31, 32]. Staff rotations and turnover reflect the dynamics of typical cancer care clinics. Regular awareness programs are thus critical to ensure that new staff is aware of the existence of these services.

Acceptance rate was relatively low across the included studies. One study, for example, found that only 64 patients took part in the exercise program out of 237 referrals received over 50 months [29]. Another study reported almost 50% rejection rate among eligible patients referred to the exercise programs [27]. In one study, some programs recorded even as low as 10% annual enrolment [32]. Service compliance was also low among those that participated in the exercise programs except for one study that recorded as high as 71% compliance over a three-year evaluation period [31]. The widely reported seemingly poor referral process may explain the low acceptance rate across the included studies. While the referral process was found to be simple and convenient for the clinicians, it may have lacked some critical elements that guaranty an effective referral mechanism, one of which is patient engagement. Illustratively, common reasons for non-utilization, missed sessions, and dropouts across the included studies were lack of interest [27], unwell due to treatment [27], COVID-19 restrictions [27], hospital readmission [27], disease progression [27], lack of awareness of service availability [29], and worsening symptoms including adverse skeletal events [31]. These experiences appear to be underlined by a general lack of exercise self-efficacy and behavioral control which is a common observation in patients on active cancer treatment [34, 35]. The poor understanding of the complex nature of cancer disease and the appropriate exercise dose with minimal adverse effects required to derive health benefits are also potential accentuating factors among these patients [34, 35]. The referral process offers the treating oncologists and nurses a unique opportunity to support their patients in building confidence in their capabilities to initiate and maintain optimal exercise behavior. Oversimplifying the referral process robs the referring clinicians of opportunities to engage proactively with and counsel patients prior to their exercise journey. The observed low service uptake further speaks to the peculiar challenges of patients on active cancer treatment, especially those on hospital admission. These individuals constantly battle with multiple treatment-related complications and are generally unwell. Offering regular counseling and psychosocial supports and adapting exercise programs to reflect individual capacities, needs, and preferences can be another useful approach to increase uptake. Even though most patients were satisfied and willing to refer others to these programs, low service uptake and high dropout rates can be improved especially in the outpatient population by addressing sources of dissatisfaction, including early morning sessions, scheduling conflicts (i.e., by enabling a more flexible programs), absence of continuation plans (i.e., by considering, perhaps, more transformative exercise programs), and parking issues (i.e., by eliminating or subsidizing parking fees) [27, 29, 32].

Issues relating to funding also pose a major threat to sustainable service integration. Even though exercise services can be delivered with less sophisticated equipment, funds are required to cover routine operational costs, including daily consumables, staff salaries, maintenances, and in some locations, rents. Most of the programs were funded through grants and private donations [27, 29, 31, 32]. These sources are largely volatile and unsustainable. In one study, the average cost to the health service per patient was AUD $1, 104 with staff salaries being the primary expense [29]. Most of the programs could not be sustained after the evaluation period, largely due to inadequate resources. For example, one study reported that two programs were closed because of lack of funds [32]. In another study, authors reported that the exercise program was restructured at the end of the evaluation period to ensure that available funds are used to cover basic operational costs [29]. Funding is a key driver of long-term service and should form primary consideration during the program planning phase. As health systems continue to grapple with limited resource allocation globally, funding challenges are even more pronounced in exercise oncology, given the pervasive misconception about rehabilitation as largely an adjunctive service. Governments, corporate sponsors, and insurance agencies are potential opportunities that could be explored for multiple funding streams [36]. More research is therefore required to confirm the greater merits of integrative exercise-cancer care models to the broader health systems. This can provoke a cultural shift in healthcare funding policies to guaranty sustainable funding for exercise-based rehabilitation.

The strength of this review is evident in our reliance on multiple implementation outcome frameworks. By drawing extensively on well-established frameworks, our findings and recommendations offer critical information to support evidence-based practice, decision making, and future research. One major limitation of the current review is the limited number of studies available for inclusion, hence the inability to weigh fully without overstating the extent to which the differences across treatment settings may have influenced service implementation and how implementation may change in other cultures/settings. Further, as per the aim of our review, we did not evaluate the potential for this approach to translate into measurable clinical benefits. By excluding non-English articles, we may have further missed out on studies that could strengthen our findings and recommendations. Oncology care models that foster integrative exercise-cancer care units are recent and largely at the evaluation stage. While this may explain the paucity of literature, we caution that the current evidence only informs decision-making and evidence-based practice in light of these limitations and individual local settings.

## Conclusion

Addressing questions around the feasibility of embedding exercise service units in clinical oncology settings is imperative for developing a sustainable exercise-oncology clinical pathway. While this appears to be an effective approach for increasing access to exercise-based rehabilitation for individuals on active cancer treatment, the current findings reveal major challenges with service penetration, adoption, and utilization. Issues relating to funding, lack of detailed implementation plan, and low organizational buy-in were the major barriers to effective service integration, particularly at the health service level. Common reasons for non-utilization, missed sessions, and dropouts were lack of interest, unwellness due to treatment, COVID-19 restrictions, hospital readmission, disease progression, lack of awareness of service availability, and adverse skeletal events.

Even though this model appears feasible for clinicians and patients, efforts are still required to drive sustainable service integration. Key priorities include seeking [local] actions to address issues relating to funding and organizational buy-in. Important considerations may include developing an implementation plan to guide the implementation process, expanding patient core management team to include staff from the exercise unit, and exploring the role of patient feedback in increasing clinician participation (e.g., treating physicians and nurses) in the referral process. Future research should consider effective strategies to promote patients’ sense of self-efficacy and behavioral control and the place of audit and feedback in improving exercise service delivery and overall service implementation. The current review recognizes the integration of exercise services with oncology care as a complex process and calls for efforts, including strategies and structures, that reflect the organizational dynamics of the clinical service environment housing the exercise unit.

## Supplementary Information

**Additional file 1.** Initial search strategy in PubMed.

## Data Availability

All data generated and analysed during this study are included in this manuscript [and its supplementary information files].

## Abbreviations

PRISMA: Preferred Reporting Items for Systematic Review and Meta-Analysis
CINAHL: Cumulative Index to Nursing and Allied Health Literature
