# Supplementary material for: A scoping review examining the integration of exercise services in clinical oncology settings
Source: BMC Health Serv Res. 2022 Feb 21;22:236. doi: 10.1186/s12913-022-07598-y (PMC8859567; doi:10.1186/s12913-022-07598-y)
Supplement: Supplementary file 1 — Additional file 1. Initial search strategy in PubMed. [file 12913_2022_7598_MOESM1_ESM.docx]

# Additional file 1

**Initial search strategy in PubMed**

| Cancer | #1 | cancer OR malignan* OR oncolog* OR neoplasm* OR tumor OR tumor OR carcinoma OR (MH "Neoplasms") OR (MH "Radiation Oncology") OR (MH "Medical Oncology") |
| --- | --- | --- |
| Exercise | #2 | exercis* OR “exercise-based rehabilitation” OR “exercise clinic” OR “exercise services” OR “exercise oncology clinical pathway” OR “exercise program*” OR (MH "Exercise Therapy") OR (MH "Exercise") |
| Outcomes | #3 | (Acceptab* Or Satisf*) OR (Adopt* OR Uptake OR utili*OR implement* OR “intention to try” OR barrier* OR enable* OR facilitate*) OR (Appropriat* OR “perceived fit” OR relevan* OR compat* OR suitab* OR useful* OR practica*) OR (cost* OR economic* OR finance*) OR (Feasibil*OR Utili* OR Practica*) OR (Fidelity OR Integrity OR “delivered as intended” OR adhere* OR “quality of program delivery”) OR (Penetrat* OR integrat* OR “spread” OR “service access”) OR (Sustain* OR maintenance OR continu* OR durab* OR incorporate*OR integrat* OR institutionaliz* OR maintain* OR routin*OR institutionalis*) OR (MH "Patient Satisfaction") OR (MH "Intention") OR (MH "Costs and Cost Analysis") OR (MH "Cost-Benefit Analysis") OR (MH "Cost Savings") OR (MH "Cost of Illness") OR (MH "Quality Control") OR (MH “Delivery of health care”) OR (MH ”Comprehensive health care”) OR (“MH “Quality of health care”) |
|  | #4 | #1 AND #2 AND #3 |
|  | #5 | #4 AND **(randomized controlled trial OR RCT OR quasi randomized controlled trial OR Quasi RCT" OR controlled clinical trial OR "controlled trial OR pretest post-test OR crossover OR cohort OR prospective OR observation* OR quantitative* OR mixed methods OR qualitative*)** |
